# Fatty Acid Profile Changes During Gradual Soil Water Depletion in Oats Suggests a Role for Jasmonates in Coping With Drought

**DOI:** 10.3389/fpls.2018.01077

**Published:** 2018-07-31

**Authors:** Javier Sánchez-Martín, Francisco J. Canales, John K. S. Tweed, Michael R. F. Lee, Diego Rubiales, Aurelio Gómez-Cadenas, Vicent Arbona, Luis A. J. Mur, Elena Prats

**Affiliations:** ^1^Institute of Sustainable Agriculture, Consejo Superior de Investigaciones Científicas (CSIC), Córdoba, Spain; ^2^Institute of Biological, Environmental and Rural Sciences, Aberystwyth University, Aberystwyth, United Kingdom; ^3^Ecofisiologia i Biotecnologia, Departament de Ciències Agràries i del Medi Natural, Universitat Jaume I, Castellón de la Plana, Spain

**Keywords:** drought, fatty-acids, jasmonates, lipids, oats, profiling

## Abstract

Although often investigated within the context of plant growth and development and/or seed composition, plant lipids have roles in responses to environment. To dissect changes in lipid and fatty acid composition linked to drought tolerance responses in oats, we performed a detailed profiling of (>90) different lipids classes during a time course of water stress. We used two oat cultivars, Flega and Patones previously characterized as susceptible and tolerant to drought, respectively. Significant differences in lipid classes (mono, di and triacylglycerols; [respectively MAG, DAG, and TAG] and free fatty acids [FFA]) and in their fatty acid (FA) composition was observed between cultivars upon drought stress. In Flega there was an increase of saturated FAs, in particular 16:0 in the DAG and TAG fractions. This led to significant lower values of the double bond index and polyunsaturated/saturated ratio in Flega compared with Patones. By contrast, Patones was characterized by the early induction of signaling-related lipids and fatty acids, such as DAGs and linolenic acid. Since the latter is a precursor of jasmonates, we investigated further changes of this signaling molecule. Targeted measurements of jasmonic acid (JA) and Ile-JA indicated early increases in the concentrations of these molecules in Patones upon drought stress whereas no changes were observed in Flega. Altogether, these data suggest a role for jasmonates and specific fatty acids in different lipid classes in coping with drought stress in oat.

## Introduction

Lipids play an important role as major energy storage compounds, as essential components of membranes and as signaling molecules (Singh et al., 2002; Vrablik and Watts, 2012). Lipids are usually classified as neutral/simple lipids and polar/complex lipids. Neutral lipids include monoacylglycerols (MAG), diacylglycerols (DAG), and triacylglycerols (TAG), consisting on a glycerol moiety with one, two or three hydroxyl groups esterified to a fatty acid (FA), respectively, and free (unesterified) fatty acids (FFA). Polar lipids (PL) are the majority class accounting for approximately 90% of total lipids and constitute 40% of membrane dry matter (Thi et al., 1990). Crucially, they contain a polar moiety but this is also linked to a glycerol backbone that may be also esterified to a variety of FAs. Thus, a particular lipid class will include numerous various molecular species differing in the nature of their FAs. The length of the acyl chains of these FAs usually ranges from 10 to 24 carbon atoms and may be fully saturated or unsaturated with double bonds at different positions. Both the length and the level of fatty acid saturation can confer distinct properties to a fatty acid. For instance, the presence of even a single double bond greatly reduces the ability of fatty acids to pack together in membranes allowing for increased fluidity (Vrablik and Watts, 2012). In addition, long chain polyunsaturated fatty acids may act as precursors for signaling molecules. For instance, linolenic acid (18:3) released from complex lipids by phospholipase activity is the precursor for phyto-oxylipin biosynthesis (Blee, 2002).

Oat (*Avena sativa*) currently ranks at around sixth in world cereal production statistics (FAO). This is due partly to the good adaptation of oat to different soil types including marginal soils where oats can perform better than other small-grain cereals (Stevens et al., 2004; Buerstmayr et al., 2007; Løes et al., 2007; Ren et al., 2007). However, oat transpiration rates and hence water requirements are higher than that of other small grain cereals (Ehlers, 1989). Thus, oats are especially susceptible to grain abortion caused by drought, which shows as empty white spikelets (Sánchez-Martín et al., 2017). Therefore, there is a need to derive oats lines with higher yields under water-limited conditions. Indeed, drought is considered the most important stress in reducing crop quality and productivity worldwide compromising both economic output and food security (Farooq et al., 2009).

Understanding plant tolerance to drought is therefore of fundamental importance and has been the object of extensive investigations over the last decade (reviewed in Osakabe et al., 2014). These have revealed part of the intricate network of genes induced upon drought stress including those involved in ABA, late embryogenesis abundant (LEA) protein, chaperone biosynthesis, those related to reducing reactive oxygen species (ROS) or ion homeostasis. Additionally, key transcription factors regulating drought-responsive gene transcription have been identified such as MYB, MYC, DREB/CBF, ABF/AREB, NAC, and WRKY (Stockinger et al., 1997; Sakuma et al., 2006; Tran et al., 2007; Nakashima et al., 2009). Similarly, drought responsive protein kinases, such as RPK1, SNF1-related protein kinase 2C, the guard cell-expressed calcium-dependent protein kinases CPK3 and CPK6 (Agrawal et al., 2003; Umezawa et al., 2004; Mori et al., 2006; Osakabe et al., 2010) have also been identified. These drought responsive features have been defined from studies in model species such as Arabidopsis. However, a number of studies show substantial dissimilarities in drought tolerance responses between model plants and crops, and even between closely related crops. Therefore, it is necessary to explore the responses leading to drought tolerance for a given species and especially in economically important crops.

From the biochemical point of view, most studies aiming to decipher drought tolerance mechanisms have focused on changes in polar metabolites and associated metabolic pathways. As a result, these studies have highlighted a role for sugar alcohols (e.g., mannitol) or sugars (e.g., raffinose family oligosaccharides), and also amino acids (e.g., proline) and amines (e.g., glycine, betaine, and polyamines). The function of these metabolites is (i) as solutes that stabilize enzymes, membranes and other cellular components, (ii) as osmolytes to reduce cellular dehydration; and (iii) as chelating agents that sequester metals and inorganic ions (Guy et al., 2008). Our own previous studies in oat revealed an important function for salicylate signaling pathways and the modulation of carbon, antioxidant and photo-oxidative metabolism (Sánchez-Martín et al., 2012, 2015). Fewer studies have attempted to discern the role of non-polar metabolites, such as lipids, in drought tolerance responses. Nevertheless, these have suggested a role for lipid metabolism in coping with drought (e.g., Gigon et al., 2004) but the underlying mechanisms are largely unknown.

In order to shed light on the role of lipids in drought tolerance in oats, we profiled more than 90 FAs for each of the main lipid classes (i.e., PLs, MAGs, DAGs, TAGs) and FFAs in two well characterized oat genotypes differing in their response to drought (Sánchez-Martín et al., 2012, 2014, 2015, 2017). Since the experimental design was also similar to our previous studies (i.e., Sánchez-Martín et al., 2015) it was possible to relate the results with previously observed changes in polar metabolites and associated biochemical pathways. These studies and targeted assays suggested a role of the production of the jasmonate phyto-oxylipin class in stress tolerance.

## Materials and Methods

### Plant Material, Growth Conditions and Sampling

All experiments were carried out with the oat cultivars (cvs) Flega and Patones, which are susceptible and tolerant to drought stress, respectively (Sánchez-Martín et al., 2012). Patones, which is well adapted to Mediterranean agroclimatic conditions, was developed by “Plant Genetic Resources Center” (INIA, Madrid, Spain), which also provided the seeds. Flega was developed by the Cereal Institute (Greece). Details of the genetic relationships between these cultivars have been previously reported in Montilla-Bascón et al. (2013).

Experiments were carried out with 3-week old seedlings in line with such studies as (Xiao et al., 2007; Hao et al., 2009; Gong et al., 2010; Sánchez-Martín et al., 2012). Experiments were carried out according to Sánchez-Martín et al. (2015): “Seedlings were grown in 0.5 L pots filled with peat: sand (3:1) in a growth chamber at 20°C, 65% relative humidity and under 12 h dark/12 h light with 250 μmol m^−2^ sec^−1^ photon flux density supplied by white fluorescent tubes (OSRAM). During growth, trays carrying the pots were watered regularly. At day 21, watering was withheld in those plants selected for drought treatment for a period of 18 days. Control plants were watered as described above throughout the whole experiment. During the drought treatment, the relative water content of the soil was monitored daily and reached approximately 20% by day 18. As previously observed (Sánchez-Martín et al., 2012, 2015), no significant differences were observed in the soil water content when growing either of the two genotypes during the drought treatment. This indicated that they were subjected to similar water stress during the whole experiment. Sampling times were chosen to cover different levels of sRWC: mild water deficit (9 daww, 40–45% sRWC), moderate water deficit (12 daww, 30–35% sRWC), high water deficit (15 daww, 20–25% sRWC) and severe water deficit (18 daww; 15–20% sRWC).”

At the set time points (9, 12, 15, and 18 days), the second leaf of each oat plant from the different cultivars and treatments was harvested from watered and droughted plants, rapidly frozen in liquid nitrogen and lyophilised. Six replicate samples per time point, cultivar and treatment were assessed. Each sample consisted of a pool of 4 leaves, each one from an independent plant. Following sampling, the plant was discarded.

### Assessment of Drought Symptoms

Assessment of drought symptoms was carried out on 10 replicates per genotype/treatment. The method of drought assessment was according to Sánchez-Martín et al. (2012, 2015). “Drought severity values were assessed daily according to a 0–5 scale where 0 = vigorous plant, with no leaves showing drought symptoms; 1 = one or two leaves (older leaves) showing slight drought symptoms in the tips (less turgor) but most leaves remain erect; 2 = several leaves showing a slight decrease in the turgor, however, most of the leaves still show no drought symptoms; 3 = leaves showing bending of the tip although the rest of the leaf remain turgid, incipient yellowing of the older leaf; 4 = all leaves showing drought symptoms including incipient wilting and/or yellowing of the older leaf; 5 = all leaves starting to appear rolled and/or shrunken. The scoring corresponds approximately to the following leaf relative water content: 1 = 80–85%; 2 = 70–75%; 3 = 55–65%; 4 = 40–50%; 5 = 30–35%. Daily visual scoring data were used to calculate the area under the drought progress curve (AUDPC) similarly to the area under the disease progress curve widely used in disease screenings (Jeger and Viljanen-Rollinson, 2001) using the formula:

AUDPC=∑ki=112[(Si+Si+1)(ti+1−ti)]

where S_i_ is the drought severity at assessment date i, t_i_ is the number of days after the first observation on assessment date i and k is the number of successive observations.

### Lipid Extraction, Fractionation and Quantification

The extraction and analyses of total fatty acids were performed as described by Kramer and Zhou (2001) and Mossoba (2001). Briefly, 100 mg of freeze-dried material were ground and 100 μL of internal standard (C23, 15 mg/mL) and 2 mL of chloroform:methanol (2:1, v/v) were added. The extracts were mixed for 5 min on an orbital shaker and were centrifuged for 5 min at 2000 rpm. The extraction procedure was repeated twice more and the supernatants were mixed. The final extract was divided in two halves and dried completely under a nitrogen stream at 50°C and subsequently stored at −20°C. One part was methylated by direct-transesterification according to Sukhija and Palmquist (1988) and used for the fatty acid profile by gas chromatography; the other part was resuspended in 1 mL of chloroform:methanol (2:1, v/v) and used for lipid fractionation as described by Nichols (1963). Fractions were subjected to a similar methylation procedure in order to determine the fatty acid profile of each fraction. Gas chromatography was performed according to Huws et al. (2011). The gas chromatograph (CP-3800, Varian, ıPalo Alto, CA, United States) was equipped with a flame ionization detector, automatic injector, split injection port and a 100 m fused silica capillary column (i.d., 0.25 mm) coated with 0.2 mm film of cyanopropyl polysiloxane (CP-Sil 88; Varian) using hydrogen as the fuel and helium as the carrier gas. The total FAME profile in a 1 ml sample at a split ratio of 1:30 was determined using a temperature gradient program described by Lee et al. (2005). Detection temperature was set at 255°C and injection temperature at 250°C. The temperature profile of the oven was as following: temperature was set at 70°C during 1 min then increased 5°C per min until 100°C, this temperature was held for 2 min, then increased by 10°C per min to 175°C, held for 34 min, increased by 4°C per min to 225°C and then held at this temperature for 29 min. The line pressure was set at 40 p.s.i. during the first 52.5 min and then increased by 0.5 p.sp.i. per min to 45 p.s.i. and held until the run finished. Peaks were identified by comparison of retention times with authentic FAME standards (ME61, Larodan fine chemicals, Malmo, Sweden; S37, Supelco, Poole, Dorset, United Kingdom) and quantified using the internal standard using the Varian star 6.4.1 software (Varian).

### Jasmonate Quantification

Jasmonate quantification was performed according to de Ollas et al. (2013). A sample of 0.4 g of frozen plant material was extracted in 5 mL of distilled water, after spiking with 100 ng dihydrojasmonic acid as internal standard. After centrifugation at 4000 *g* at 4°C, supernatants were recovered and pH adjusted to 3.0 with 30% acetic acid. The acidified water extract was partitioned twice against 3 mL of di-ethyl ether. The organic layer was recovered and evaporated under vacuum in a centrifuge concentrator. The dry residue was then resuspended in 1 mL of a 10% MeOH solution by gentle sonication. The resulting solution was filtered and directly injected into a HPLC system (Acquity SDS UPLC, Waters Corp., Milford, MA, United States). Separations were carried out on a C18 column (C18 Gravity, 1.8 μm particle size, 50 × 2.1 mm, Macherey-Nagel, Germany) using a linear gradient of MeOH and water supplemented with 0.1% acetic acid at a flow rate of 300 μl min^−1^. Jasmonates (12-oxophytodienoic acid, OPDA; jasmonic acid, JA and jasmonoyl isoleucine, JA-Ile) were quantified with a TQS triple quadrupole mass spectrometer (Micromass Ltd., Manchester, United Kingdom) connected online to the output of the column through an orthogonal Z-spray electrospray ion source. Transitions for JA/DHJA (209 > 59/211 > 59), OPDA (291 > 165), and JA-Ile (322 > 130) were monitored in negative ionization mode. Quantitation of plant hormones was carried out by external calibration using standards of known concentration. Processing of chromatograms, integration of peaks and quantitation was performed with Masslynx 4.1 software (Micromass Ltd., Manchester, United Kingdom).

### Data Analysis

All experiments were performed in completely randomized designs. For ease of understanding, means of raw percentage data were presented in Tables and Figures. However, for statistical analysis, data recorded as percentages were transformed to arcsine square roots to normalize data and stabilize variances before being subjected to analysis of variance using SPSS software. Afterward, residual plots were inspected to confirm data conformed to normality. Shapiro–Wilk test and Bartlett’s test was performed to test normality and homogeneity of variances respectively. Significance of differences between means was determined by LSD and contrast analysis (Scheffe’s). Linear regression analyses were used for calibration of all analyzed metabolites. Calibration curves were described by the following linear equation: *y* = *ax* + *b*, where *y* is the metabolite area and *x* is the concentration. The slope, intercept and correlation coefficient were calculated for each regression curve. The limits of detection (LOD) and limit of quantitation (LOQ) were determined based on the linear regression across the concentration range used for calibration. LOD and LOQ were calculated as the ratio *3S_a_/b and 10S_a_/b*, respectively, where *S_a_* is the standard deviation of the response and *b* is the slope of the calibration curve. For multivariate analyses, the data were analyzed using canonical analysis.

## Results

As expected (Sánchez-Martín et al., 2012, 2015) cv. Flega was susceptible and Patones resistant to drought. Flega showed earlier drought symptoms than Patones starting as a slight loss of turgor in the tip of the older leaves and then progressing down the leaf and into younger leaves as soil water content decreased. This was accompanied by a yellowing of the older leaves at the latter stages of water deficit. Thus, by the end of the experiment, Flega plants were scored at a 5 value and showed drought associated senescence in the first leaves, whereas Patones plants did not reach a score of 4 and were visually healthier than Flega (Sánchez-Martín et al., 2015). Importantly, even in Flega, plants were still far from the wilting point which usually occurred > 10 days later at c.a. 6–9% sRWC.

### Total Lipid and Lipid Fraction Contents

**FIGURE 1:**
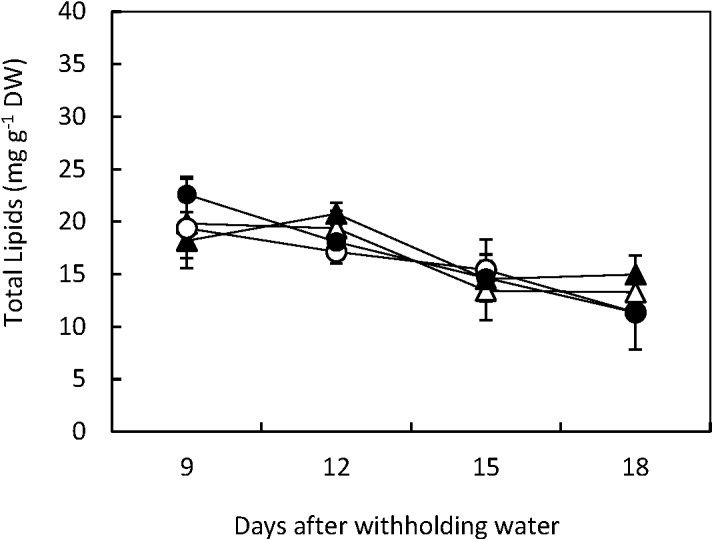
Total Lipids. Total lipids were quantified in drought susceptible Flega (triangles) and resistant Patones (circles) well-watered plants (open symbols) and during a time course of water stress (solid symbols) (9, 12, 15, and 18 days). Data are mean of five replicates ± standard error.

In both, Flega and Patones total lipid content was significantly (*p* < 0.001) reduced during the 18 days experimental period. However, no significant differences were observed between treatments or genotypes indicating that the lesser lipid content was related to the developmental changes in the leaves that were common to both genotypes (**Figure 1**).

**FIGURE 2:**
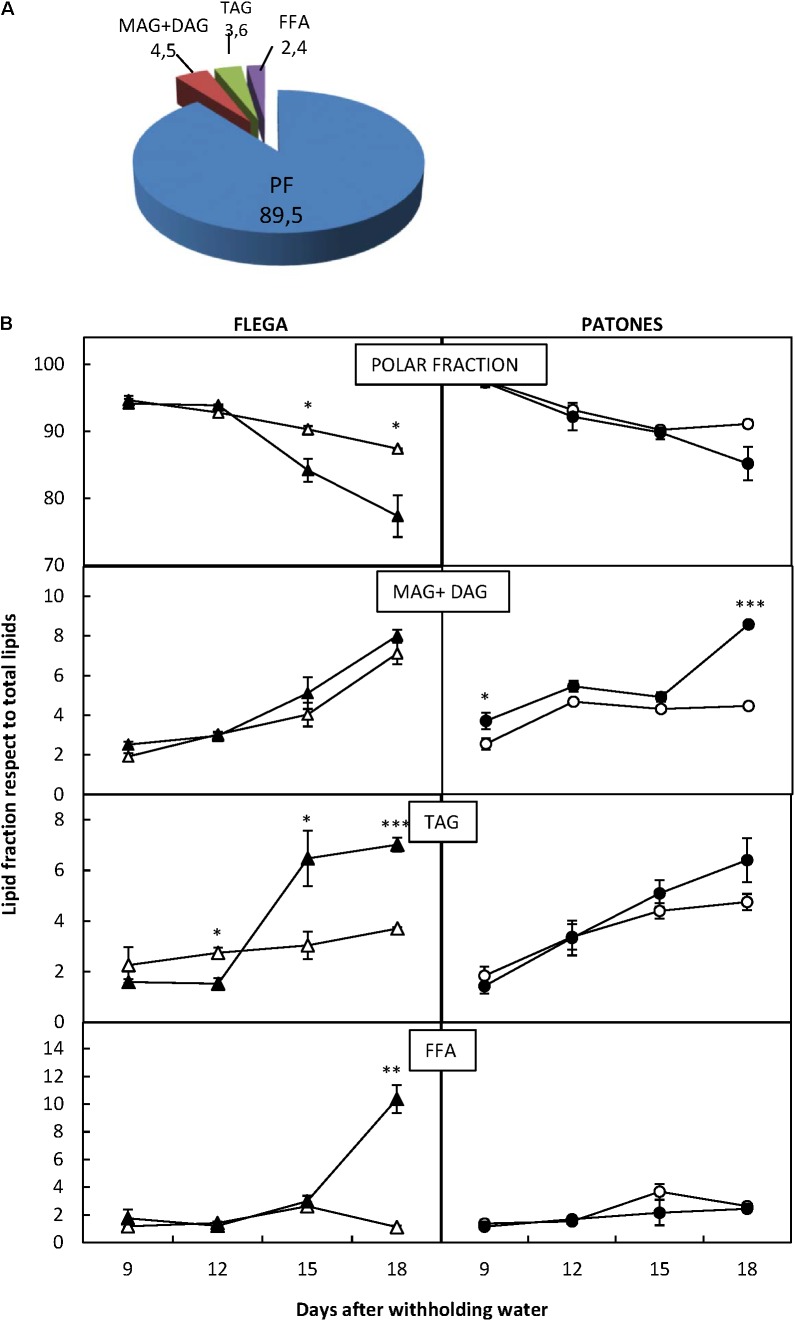
Lipid fractions. **(A)** Lipid fraction distribution. Total lipids were separated cromatographycally in four lipid fractions: polar (PF), mono and di-acylglicerides (MAG + DAG), triacylglicerides (TAG) and free fatty acids (FFA) and their proportions calculated. **(B)** Lipid Fraction dynamic over a drought time course. Each lipid fraction was quantified in drought susceptible Flega (triangles) and resistant Patones (circles) well-watered plants (open symbols) and during a time course of water stress (solid symbols) (9, 12, 15, and 18 days). Data are mean of five replicates ± standard error. ^∗^, ^∗∗^, and ^∗∗∗^ indicate significant differences at *P* < 0.05, 0.01, and 0.001, respectively.

Overall, the polar fractions contained by far the most lipids accounting for nearly 90% of the total lipids. This was followed by the fractions containing MAG+DAG (4.5%) and TAG (3.6%) and finally the FFA fraction with less than 2.5% (**Figure 2A**). These relative proportions were similar for both genotypes. However, when each of the fractions was analyzed in detail, significant differences were found with treatment and sampling time (**Figure 2B**).

Polar fraction content did not differ significantly between Flega and Patones under well-watered conditions. However, when exposed to drought, a significant reduction of PLs compared to control plants was observed in Flega from 15 days after withholding water (daww; *P* < 0.001) reaching more than 15% reduction at 18 daww. In contrast, in Patones, there was a slight but insignificant loss of PLs. MAG and DAG accumulation patterns in Flega and Patones were also different. Whereas no significant differences were observed between droughted Flega and control plants, in Patones plants both MAG and DAG increased very early (6 daww) reaching a twofold increase at the latest sampling time, 18 daww (**Figure 2B**). However, the content of TAG increased earlier and higher in Flega plants under drought than in Patones with drought (**Figure 2B**). Considering the FFA fraction, no differences were seen in Patones plants exposed to drought compared to controls, and only a very late, albeit dramatic, increase at 18 daww was observed in Flega (**Figure 2B**)

### Fatty Acid Profiling Within Lipid Fractions

#### Polar Fraction

**FIGURE 3:**
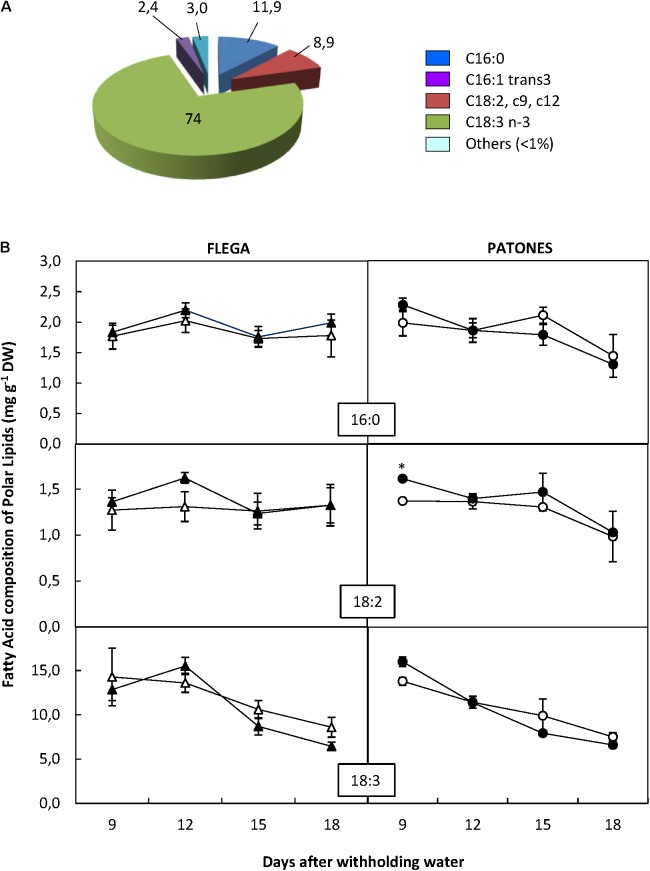
Fatty acid content of Polar fraction. **(A)** Fatty acid distribution within polar fraction. The proportion of fatty acids detected in the polar fraction was calculated from a profiling of 92 fatty acids. **(B)** Fatty acid content. Fatty acids accounting for at least 5% of the fraction were quantified in drought susceptible Flega (triangles) and resistant Patones (circles) well-watered plants (open symbols) and during a time course of water stress (solid symbols) (9, 12, 15, and 18 days). Data are mean of five replicates ± standard error. ^∗^, indicate significant differences at *P* < 0.05.

A more detailed analysis of the content of the different FA components within each of the different lipid fractions showed that out of the 92 FAs analyzed only 19 were detected in the oats (Supplementary Table 1). The polar fraction possessed the widest range of FAs (Supplementary Table 1) although most of them were in a very low proportion (<1% of the fraction) and exhibited no differences between genotypes or treatment. Only three FA exceeded 5%, C18:3 n3 (linolenic acid) being the most abundant at 74% of the fraction, followed by C16:0 (palmitic acid) at 11.9% and C18:2 cis9 cis12 (linoleic) with 8.9% (**Figure 3A**). Droughted Patones but not Flega plants slightly increased the content of these major FA in the polar fractions compared to well-watered controls at the earliest time-points assessed. At latter times points (15 and 18 daww) both Flega and Patones plants tended to decrease the content in linolenic acid (**Figure 3B**). The effect of the drought stress in the content of the other minor FA of this and the other fractions are available in Supplementary Figure 1 as a heat map. These did not show any significant differences with drought.

#### Mono- and Di-acylglyceride Fraction

**FIGURE 4:**
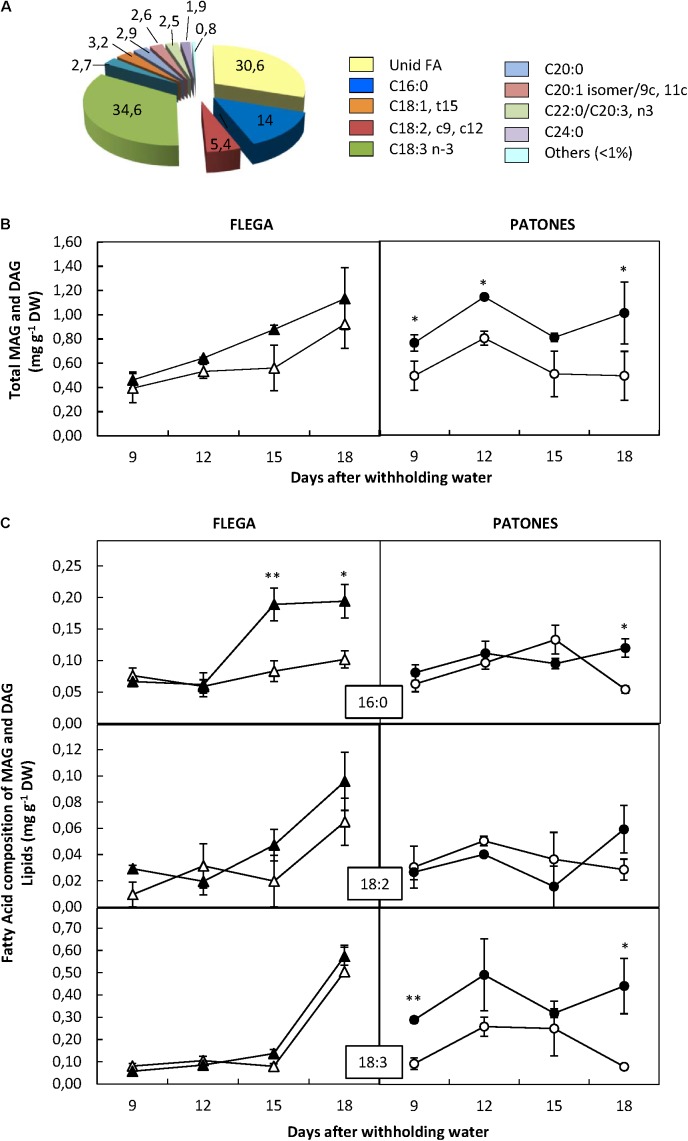
Fatty acid content of mono- and di-acylgliceride fraction. **(A)** Fatty acid distribution within mono- and di-acylgliceride fraction. The proportion of fatty acids detected in the fraction was calculated from a profiling of 92 fatty acids. **(B)** Fatty acid content. **(C)** Fatty acids accounting for at least 5% of the fraction were quantified in drought susceptible Flega (triangles) and resistant Patones (circles) well-watered plants (open symbols) and during a time course of water stress (solid symbols) (9, 12, 15, and 18 days). Data are mean of five replicates ± standard error. ^∗^ and ^∗∗^ indicate significant differences at *P* < 0.05 and 0.01, respectively.

Only 9 FA out of the 92 assessed were detected in the MAG and DAG fractions. From these, three FA exceed 5% of the total in this fraction being linolenic acid (the most abundant FA of this lipid fraction), followed by palmitic and linoleic acid (**Figure 4A**). This was similar to the accumulation patterns observed in the PF. Interestingly an unidentified FA accounting by more than 30% was observed within the MAG and DAG fraction. However, no significant differences were observed between genotypes and treatment regarding this FA.

The total content of the FA of this fraction significantly increased in cv. Patones under drought compared to the well-watered controls (*P* < 0.001) from the earliest time-point assessed. Flega plants followed a similar trend but the differences were not significant between droughted and well-watered plants (**Figure 4B**). Analysis of the MAG and DAG fractions showed a significant increase of palmitic acid (C16:0) in Flega plants under drought with respect to their controls at 15 and 18 daww (**Figure 4C**). Such an increase was observed in Patones plants only at the latest time point and not as high as in Flega (**Figure 4C**). Contrary to this, Patones plants showed significant increases of linolenic acid (C18:3) at most time points of the drought time course, which were not observed in Flega. No significant differences were observed in linoleic acid (C18:2) in any genotype or with any treatments.

#### Triacylglyceride Fraction

**FIGURE 5:**
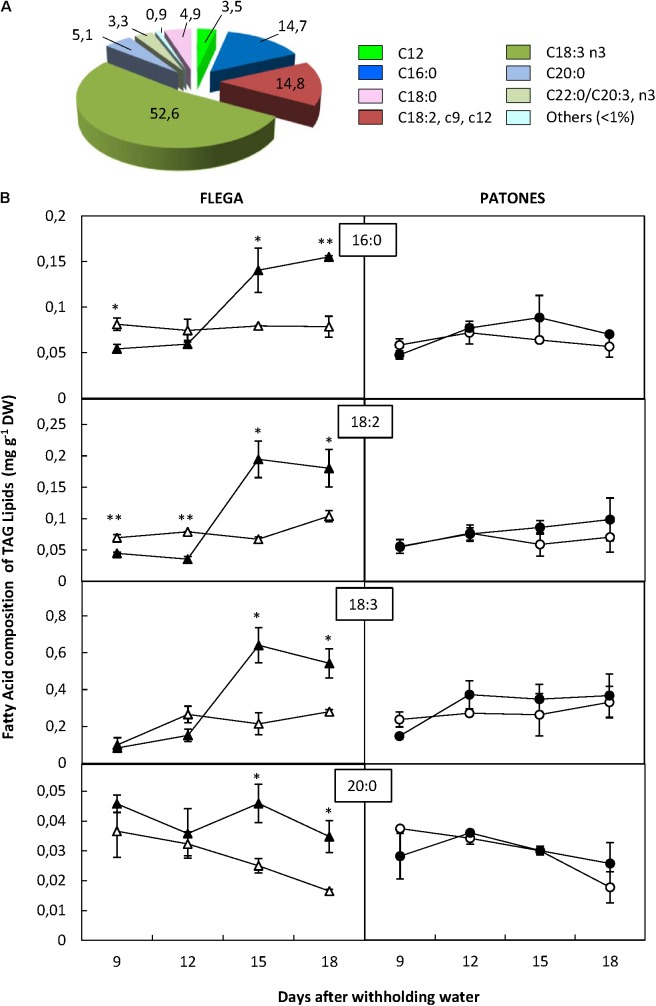
Fatty acid content of triacylgliceride fraction. **(A)** Fatty acid distribution within triacylgliceride fraction. The proportion of fatty acids detected in the fraction was calculated from a profiling of 92 fatty acids. **(B)** Fatty acid content. Fatty acids accounting for at least 5% of the fraction were quantified in drought susceptible Flega (triangles) and resistant Patones (circles) well-watered plants (open symbols) and during a time course of water stress (solid symbols) (9, 12, 15, and 18 days). Data are mean of five replicates ± standard error. ^∗^ and ^∗∗^ indicate significant differences at *P* < 0.05 and 0.01, respectively.

Similar to PF and MAG + DAG fractions, the most abundant FA of the TAG fraction were palmitic, linoleic and linolenic acids. However, in the TAG fraction, arachidic fatty acid (C20:0) detected accounted for 5.1% of the fraction and another saturated FA, stearic acid (C18:0), accounted for 4.9% of total TAGs (**Figure 5A**).

Overall, no significant differences in FA content of TAG fraction were observed in Patones plants under drought compared to their controls. However, important changes were observed in Flega. This genotype had an early (9 and 12 daww) and slight decrease of palmitic and linoleic acids followed by a dramatic increase at 15 and 18 daww of all the majority FAs of this fraction with palmitic acid increasing by 52% (*P* < 0.001), linoleic acid by 52.6% (*P* = 0.002), linolenic acid by 58.2% (*P* < 0.001) and arachidic acid by 47.5% (*P* = 0.006) (**Figure 5B**). Stearic acid followed a similar trend to that of arachidic acid (data not shown).

#### Free Fatty Acid Fraction

**FIGURE 6:**
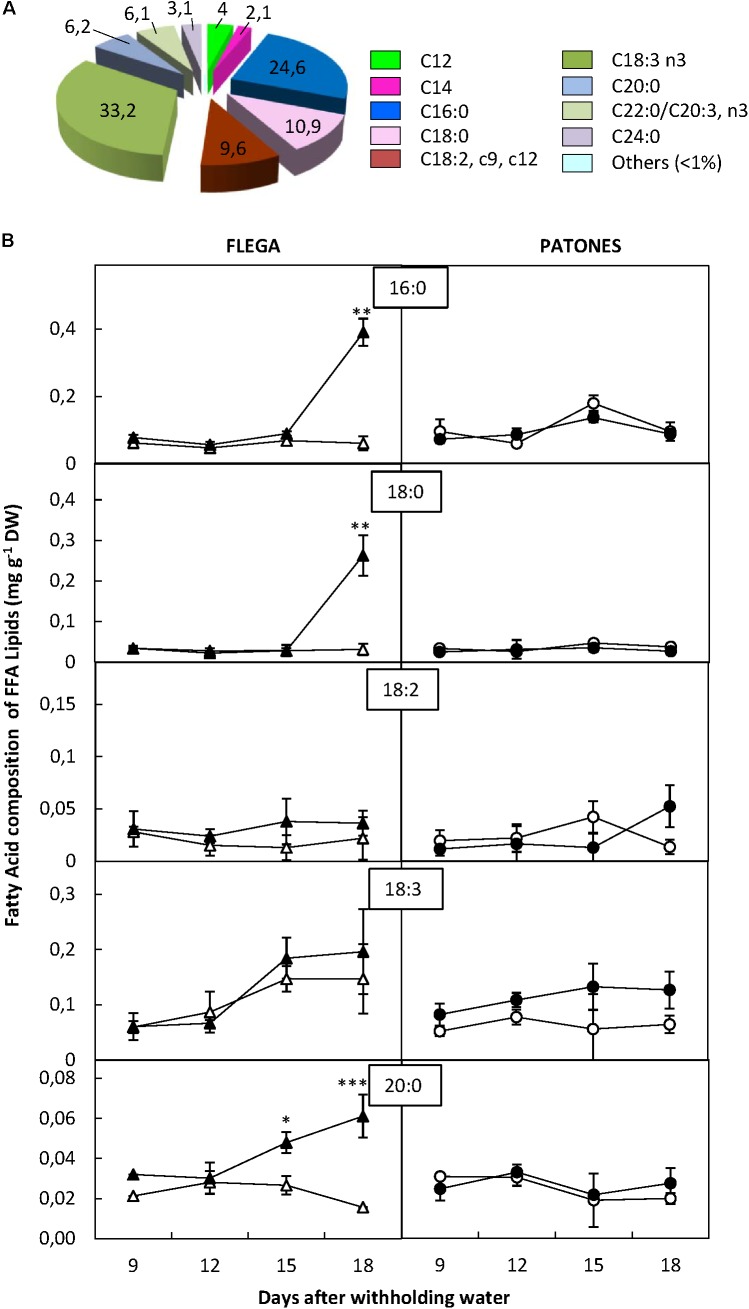
Fatty acid content of free fatty acid fraction. **(A)** Fatty acid distribution within free fatty acid fraction. The proportion of fatty acids detected in the fraction was calculated from a profiling of 92 fatty acids. **(B)** Fatty acid content. Four major fatty acid of the fraction were quantified in drought susceptible Flega (triangles) and resistant Patones (circles) well-watered plants (open symbols) and during a time course of water stress (solid symbols) (9, 12, 15, and 18 days). Data are mean of five replicates ± standard error. ^∗^, ^∗∗^, and ^∗∗∗^ indicate significant differences at *P* < 0.05, 0.01, and 0.001, respectively.

Unlike the other fractions, in the FFA fraction, the proportions of all FAs detected were higher than 1% (**Figure 6**). The most abundant FAs of the FFA fraction were linolenic (33.2%), palmitic (24.6%), stearic (10.9%) and linoleic (9.6%) acids. Two additional FAs, arachidic and eicosatrienoic and/or behenic acids were also detected accounting for more than 5% of the total fraction (**Figure 6A**). Since it was not possible to distinguish between eicosatrienoic and behenic acids, they were not included in the analysis.

Analysis of the FFA fraction showed increases in Flega droughted plants of up to 85% for the saturated palmitic, stearic and arachidic acids, compared to controls at the latest points of the drought time course (**Figure 6B**). Interestingly, the stearic acid and other saturated FAs in the other fractions also increased differentially in Flega plants under drought (Supplementary Figure 1). This increase in the saturated fatty acids of the FFA fraction was not observed in Patones.

### Overview of FA Changes Linked to Drought Tolerance

**FIGURE 7:**
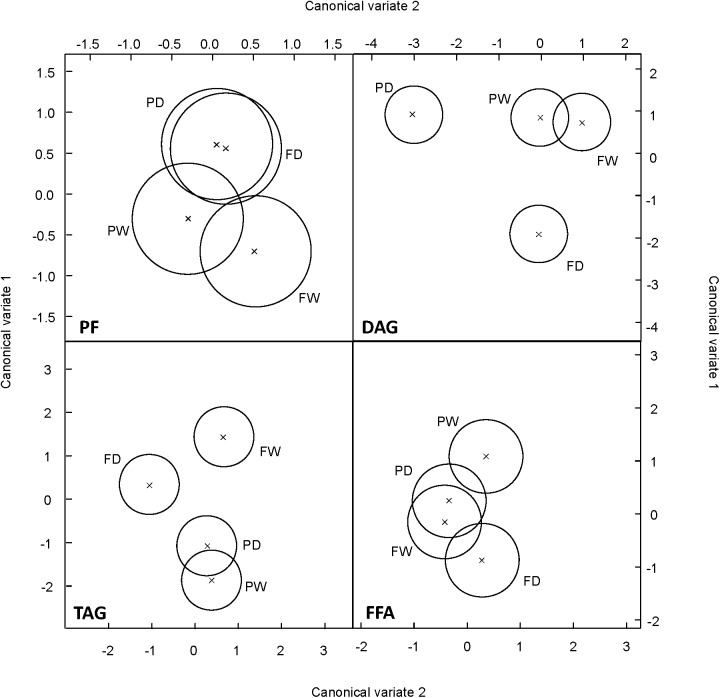
Multivariate analysis of susceptible and resistant oat genotypes according to the different fatty acids assessed. Scatterplot of Canonical variates analysis scores of components 1 and 2 based on the profile of fatty acids within each of the different lipid fractions were performed in drought susceptible Flega and resistant Patones well-watered plants (FW and PW, respectively) and during a time course of water stress (FD and PD, respectively).

In order to determine the most important changes discriminating the behavior of the two oat cvs during the drought time course, FA profiles were subjected to canonical variate analysis (CVA). This analysis showed that MAG + DAG and TAG fraction discriminated the response of the two oat genotypes under drought (**Figure 7**). Interestingly, FAs of the MAG+DAG fraction did not discriminate between both genotypes under well-watered conditions but only under drought conditions. CVA of the TAG fraction showed that while Flega plants under drought differentiated from the well-watered controls, no significant differences were observed in resistant Patones plants (**Figure 7**).

Again to provide an overview of FA changes the Double Bond Indices (DBI) and Polyunsaturated-Saturated (PUFA/SFA) ratios were calculated for Flega and Patones with each treatment. Overall, the watered controls of both genotypes showed similar DBI and PUFA/SFA ratios. However, when focusing on the changes of the droughted plants compared to their controls, important differences between genotypes arose. DBI and PUFA/SFA ratio in the PF decreased in Flega and Patones plants under drought with respect to their controls, albeit this decrease started earlier in Flega than in Patones (**Table 1**). The DAG fraction showed the largest differences between genotypes (*P* < 0.006). Compared to controls, DAGs in droughted Flega plants showed reduced DBI and PUFA/SFA ratios of approximately 15 and 10% respectively, but in Patones plants exhibited increases in both DBI and PUFA/SFA ratios of approximately 45%. A similar increase was also observed in DBI and PUFA/SFA ratio of the TAG and FFA fraction in Patones whereas Flega showed decreases of both parameters at several time points in DAG, TAG and FFA fraction with a marked increase at 15 daww in the TAG fraction. Interestingly, linolenic acid, which was the major fatty acid in all fractions, were positively correlated with DBI for the polar (*r* = 0.75; *P* < 0.01) and negatively correlated for the TAG (*r* = −0.59; *P* < 0.05) fractions in plants subjected to drought whereas no correlation were observed in well-watered controls plants.

**Table 1:** Double bond index (DBI) and ratio between polyunsaturated and saturated fatty acid (PUFA/SFA) for polar lipid fraction (PF), diacylglycerides (DAG), triacylglycerides (TAG) and free fatty acids (FFA) in oat genotypes Flega (susceptible) and Patones (tolerant) during a time course of increasing water stress at 9, 12, 15, and 18 days after withholding water (daww).

Genotype	daww	PF	DAG	TAG	FFA

	Double Bond Index (DBI) compared to watered controls				
Flega	9	99,6 ± 0.6	79,6 ± 17.5	98,3 ± 1.4	133,5 ± 0.03^∗^
	12	99,8 ± 0.7	75,3 ± 8.0^∗^	91,3 ± 12.9	96,5 ± 1.3
	15	97,3 ± 0.1^∗∗^	93.3 ± 24.7	128,8 ± 1.3^∗^	100,9 ± 8.4
	18	91,9 ± 3.5^∗^	74,0 ± 10.0^∗^	91,7 ± 7.1	70,1 ± 11.5
Patones	9	99,9 ± 0.6	146,6 ± 7.3^∗^	89,9 ± 14.6	153,3 ± 5.8^∗^
	12	101,5 ± 0.3	113,0 ± 9.8	106,2 ± 0.8^∗^	117,2 ± 9.8
	15	101,0 ± 0.6	101,5 ± 14.4	115,3 ± 0.4^∗∗^	103,5 ± 8.7
	18	94,1 ± 1.7^∗^	233,5 ± 16.4^∗∗^	103,2 ± 3.9	177,5 ± 23.8^∗^
lsd		3.194	42.2	15.63	25.64

	**PUFA/SFA**				

Flega	9	101,3 ± 4.3	108,1 ± 33.8	71.68 ± 1.31^∗^	142,3 ± 56.1
	12	101,6 ± 4.8	93,3 ± 27.4	110,3 ± 23.8	83,8 ± 5.35^∗∗^
	15	85,1 ± 2.5^∗∗^	119,1 ± 42.8	197,0 ± 4.2^∗∗^	184,77 ± 29.62
	18	72,6 ± 9.9^∗^	40,1 ± 7.7^∗∗^	75,0 ± 16.7	125,99 ± 35.4
Patones	9	100,1 ± 3.0	166,6 ± 10.2^∗^	105.62 ± 3.6	135,6 ± 19.0^∗^
	12	109,5 ± 2.5	147,0 ± 27.4^∗^	156,3 ± 11.2^∗∗^	127,7 ± 33.9
	15	99,1 ± 2.9	116,1 ± 17.3	134,8 ± 0.2^∗^	99,5 ± 4.0
	18	82,5 ± 5.6^∗^	235.4 ± 50.7^∗∗^	126,3 ± 12.8	250,5 ± 48.1^∗^
lsd		11.59	44.0	57.7	53.9

*Data are mean of 4 replications ± standard error. ^∗^ and ^∗∗^ indicate significant differences at P < 0.05 and 0.01, respectively, respect to their well-watered controls. l.s.d for P < 0.05 is also given for other comparisons within the table.*

### Linolenic Acid and Jasmonates

**FIGURE 8:**
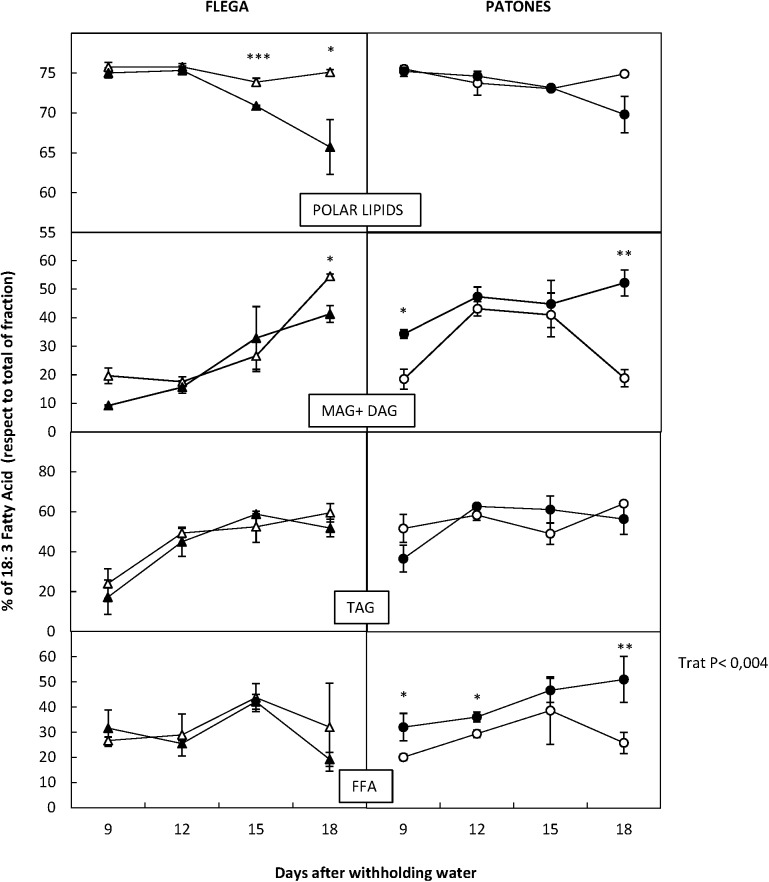
Dynamic of 18:3 fatty acids during drought respect total lipid fraction. Linolenic acid was quantified in drought susceptible Flega (triangles) and resistant Patones (circles) well-watered plants (open symbols) and during a time course of water stress (solid symbols) (9, 12, 15, and 18 days). Data are mean of five replicates ± standard error. ^∗^, ^∗∗^, and ^∗∗∗^ indicate significant differences at *P* < 0.05, 0.01, and 0.001, respectively.

The detailed profile of FAs described above suggested that there were interconversions of the lipids between different fractions and in particular in the FA profile within fractions. The most important change that discriminated between drought susceptible Flega and resistant Patones related to linolenic acid. Thus, when values were referred as percentage of the total fraction we observed that the susceptible Flega tended to reduce the proportion of linolenic acid of the polar fraction during the drought time course whereas Patones showed relative increases of linolenic acid all over the drought time course in the MAG + DAG and FFA fraction (*P* < 0.02 and 0.004, respectively) (**Figure 8**).

**FIGURE 9:**
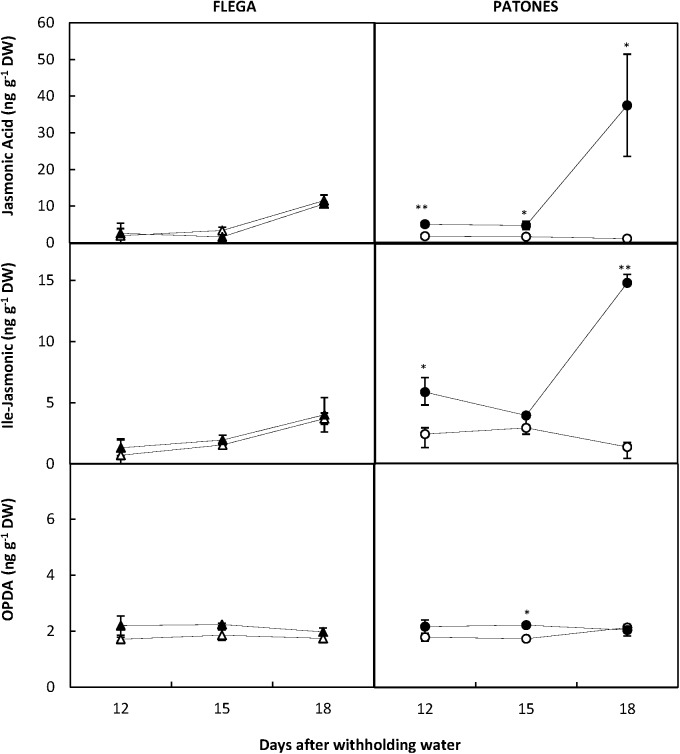
Jasmonate content during a drought time course. Jasmonic acid, Ile-jasmonic and OPDA were quantified in drought susceptible Flega (triangles) and resistant Patones (circles) well-watered plants (open symbols) and during a time course of water stress (solid symbols) (9, 12, 15, and 18 days). Data are mean of five replicates ± standard error. ^∗^ and ^∗∗^ indicate significant differences at *P* < 0.05 and 0.01, respectively.

Since linolenic acid is the precursor of the JAs, which are involved in signaling during stress responses, we measured changes in JA, the isoleucine (Ile) conjugate of jasmonic acid (JA-Ile) and the JA precursor 12-oxophytodienoic acid (OPDA). Interestingly, JA and Ile-JA increased significantly in Patones leaves compared to well-watered controls during the drought time course (*P* < 0.05 and 0.001, respectively) (**Figure 9**). However, no changes in jasmonates were observed in Flega. This correlation suggested a role for jasmonate in coping with drought stress.

Interestingly no correlation was observed in well-watered plants between JA and DBI. However, under drought conditions there was a positive correlation between JA and DBI albeit only in the DAG fraction (*r* = 0.66; *P* < 0.01). Similarly, no correlation was found between JA and linolenic acid in well-watered plants but a positive correlation were observed for these metabolites in the DAG fraction (*r* = 0.60; *P* < 0.05) following drought. This suggests a drought induction of the pathway leading to linolenic acid in the DAG fraction and JA biosynthesis.

## Discussion

It is now accepted that FAs and lipids are more than simply storage compounds or structural components of membranes. They also regulate processes such as growth and development and responses to biotic and abiotic stresses for acclimation (Upchurch, 2008). Our previous metabolomic studies on drought tolerance in oats have focused in polar metabolites (Sánchez-Martín et al., 2015), but clearly, the potential roles of lipids and FAs should also be considered.

Considered as a whole, we did not observe differences in the total lipid content between the susceptible and resistant oat genotypes or between the drought-stressed plants compared to their well-watered controls. During the course of the drought experiments a slight reduction in total lipids was observed in all genotypes and treatments, probably reflecting the process of slowly natural leaf senescence during the 40 days period of the experiment. The oxidation and release of membrane fatty acids are known to be involved in senescence process (Chapman, 1998).

More detailed analyses showed differences in each of the lipid classes and their FAs. These showed significant differences between the drought responses of resistant and susceptible genotypes. Interestingly, the observed patterns appeared consistent with a re-distribution of already existing FAs to give different profiles. Thus, in Flega the dramatic reduction in PL correlated well with increases in TAG and FFA, whereas in Patones the slightly reduction in PL under severe stress correlated with an increase in DAG at this time point. There were slight increases of MAG+DAG over the whole time-course in Patones in absolute values (**Figure 4**); possibly indicating the biosynthesis of minority neutral lipid classes.

The dramatic reduction of PL observed in Flega during the drought time-course could reflect membrane damage that would agree with previous studies that reported an inhibition of lipid biosynthesis under drought stress (Thi et al., 1990; Depaula et al., 1993). This decrease in the PL of Flega leaves during the drought time-course correlated with our previously reported increases in lipolytic activities (Sánchez-Martín et al., 2012). This may lead to losses in cell compartmentation and protein function (Gigon et al., 2004). Such changes in the composition of the lipid bilayer would influence lipid-protein and protein–protein associations, membrane-bound enzyme activities and the carrier-mediated transport capacity of membranes which are essential for cell division, biological reproduction and intracellular membrane trafficking (Quartacci et al., 2002). It would be expected that in stressed Flega plants the reduction in polar lipids would severely impair such functions, a feature not observed in the resistant genotype until severe water deficit. Beyond this, cell membranes are also the first receptors of stress achieved through quantitative changes in the unsaturation level of the membrane fatty acids (van Meer et al., 2008). These can have two effects, altering the rigidity of the cell structure and also associated signaling events.

Our data, taken along a relatively long period of increasing water deficit, report a sharp decrease in linolenic acid of the polar fraction in the sensitive accession at moderate water stress. This contrasts with the resistant genotype Patones where levels of linolenic acid stay constant for most time points and only slightly decreases at severe stress could be observed. The reduction in 18:3 levels in Flega correlated with an earlier decrease in the double bond index and PUFA/SFA ratio. Linolenic acid is the major fatty acid of PLs. In the case of *Avena*, it accounts for approximately 74% of total PLs and is mainly concentrated in the chloroplast membranes. Linolenic acid is crucial to maintain membrane integrity and the functionality of integral membrane proteins, such as the proteins forming the photosynthetic machinery hence, its concentration decrease has a major impact on photosynthesis (Upchurch, 2008). In addition, the degradation of linolenic acid and other polar lipids release FFAs and lipid hydroperoxides, which trigger the senescence process (Leshem et al., 1986). Interestingly, a reduction in photosynthesis and the induction of senescence symptoms can be observed in Flega at moderate drought stress, which are not observable in the resistant Patones (Sánchez-Martín et al., 2015).

Patones showed significant increases in the content of linolenic acid within the MAG + DAG pool and also in the FFA fraction (**Figure 8**) whereas in Flega the levels of this compound did not change or even reduced. Linolenic acid is the precursor of jasmonates (JAs), important signaling molecules involved, among other processes, in stress resistance. Although a role for JAs in the adaptation to drought stress has been suggested, the molecular mechanisms of the role of JAs in drought stress-signaling are still mostly unclear (Riemann et al., 2015). Indeed, JAs have been reported to improve drought tolerance in some studies but others suggest that it causes a reduction in growth and yield. These differences could depend on the type of plant and tissue assessed, intensity and duration of drought stress and, where exogenous applications are performed, on the JA dosage applied (Kim et al., 2009). Our results show an increase of free linolenic acid in the resistant genotype from the earliest sampling time assessed. This increase was associated with JA and Ile-JA accumulation but not its biosynthetic intermediate 12-OPDA. The increase in free linolenic acid in Patones was at least 15-fold excess over that required to account for the levels of newly synthesized JA (Conconi et al., 1996). Despite this considerable increase, in absolute levels it is a minor proportion of membrane lipids so, in agreement with previous reports, JA increases do not result in a change in total lipid content (Conconi et al., 1996). JAs could play an important role in signaling drought-induced antioxidant responses, in particular related to ascorbate metabolism (Ai et al., 2008), which is a feature of Patones plants under drought (Sánchez-Martín et al., 2015). In addition to changes in linolenic acid, lipoxygenase activity (LOX) is also linked to jasmonate biosynthesis but also to lipid peroxidation. Whereas no differences in LOX activity has been described in well-watered Flega and Patones plants a significant increase in LOX activity in water-stressed plants were observed (Sánchez-Martín et al., 2012). Interestingly, while there was a significant correlation between the level of LOX activity and lipid peroxidation in susceptible Flega, this correlation was not found in the resistant Patones. Overall, the pattern of early free linolenic acid and jasmonates accumulation in Patones plants and the lack of correlation with lipid peroxidation suggests a role for these signaling molecules driving the resistant response.

As stated above, the dramatic reduction of polar lipids in Flega was accompanied by an increase in TAG. The formation of TAG, as a result of membrane lipid hydrolysis have been observed previously during drought stress for desert shrubs (Benadjaoud et al., 2013), forage grasses (Perlikowski et al., 2016), a resurrection plant (Gasulla et al., 2013), also in crop species (Thi et al., 1990) and as a result of other abiotic stresses (Sakaki et al., 1990). In contrast to the membrane functions of PLs, the functional significance of TAGs in vegetative tissues has not been fully described. It has been suggested that the accumulation of TAG might be a way of regulating the levels of FFA, which disrupt a range of membrane functions (Mckersie et al., 1988; Singh et al., 2002). This might explain the effects of the substantial FFA accumulation at the latest sampling time of the drought time-course in Flega which could cause an unbalance between the increase of PLs released from the membrane and the capacity to esterify the resulting FFA to TAG. Alternatively, the accumulation of TAG is a mechanism for dissipation of excess radiation energy in leaves that is induced at increased levels of photoinhibition (Marchin et al., 2017). The rationale of this hypothesis is that the production of the energy-rich reduced carbon compounds requires about twice the energy of carbohydrate biosynthesis (Solovchenko, 2012). This fits well with our observations since from 15 daww the level of photoinhibition was significantly increased in Flega (Sánchez-Martín et al., 2015).

With Patones and not Flega, we observed a rapid increase in DAG from the earliest sampling time assessed and all along the stress period. DAG is an important signaling molecule in animal cells, binding to the C1 domain of key signaling proteins as protein kinase C and activating them (Munnik and Testerink, 2009). In plants, phosphatidic acid (PA; the phosphorylated product of DAG) rather than DAG itself has been most often implicated as a major secondary messenger (Munnik and Testerink, 2009). However, DAG itself may act as a signaling molecule during plant development and in response to certain environmental stimuli (Dong et al., 2012). For example, progressive dehydration in *Vigna unguiculata* led to the accumulation of the transcripts of two PA phosphatases genes, involved in the catalysis from PA to DAG (Franca et al., 2008). Irrespective of the involvement of DAG itself or PA, the fast increase in this lipid class suggests early signaling events in Patones leading to processes that avoid drought-related damage.

Our previous reports, suggest that in Flega, rapid stomatal closure, a later induction or low photorespiration and a weak induction of antioxidant pathways lead to an increase in ROS, damaging the photosynthetic apparatus, and reducing cell membrane stability (Sánchez-Martín et al., 2015). Here, we extend this study to show that this decrease of the cell membrane stability is supported by the decrease in membrane lipids observed together with the increase in saturated FA and a reduction of the double bond index and PUFA/SFA ratio. This can be related to the observed triggering of drought-induced senescence. Accumulation of TAG could be related to a survival response in this genotype in an attempt to reduce the levels of free fatty acids and hence damage. By contrast, Patones is characterized by the fast induction of metabolic responses suggesting either a lower stress threshold or faster drought sensing for triggering a response (Sánchez-Martín et al., 2015). This was also reflected in the early induction of signalisation related lipids and fatty acids, such as DAGs and linolenic acid and their related JAs derivatives driving processes probably related to impair oxidative stress.

## Author Contributions

JS-M and FC made most of the experimental work and data analysis. DR contributed to the stress resistance aspects. JT and ML contributed to the fatty acid profiling aspects. LM supervised the fatty acid profiling experiments. AG-C and VA contributed to the jasmonate sections. EP steered the research, designed the experiments, and contributed to the interpretation of results and writing of the manuscript. All authors also contributed to critical reading and writing.

## Conflict of Interest Statement

The authors declare that the research was conducted in the absence of any commercial or financial relationships that could be construed as a potential conflict of interest.

## References

[B1] Agrawal G. K. Tamogami S. Iwahashi H. Agrawal V. P. Rakwal R. (2003). Transient regulation of jasmonic acid-inducible rice MAP kinase gene (OsBWMK1) by diverse biotic and abiotic stresses. *Plant Physiol. Biochem.* 41 355–361. 10.1016/s0981-9428(03)00030-5

[B2] Ai L. Li Z. H. Xie Z. X. Tian X. L. Eneji A. E. Duan L. S. (2008). Coronatine alleviates polyethylene glycol-induced water stress in two rice (*Oryza sativa* L.) cultivars. *J. Agron. Crop Sci.* 194 360–368. 10.1111/j.1439-037X.2008.00325.x

[B3] Benadjaoud A. Benhassaine-Kesri G. Zachowski A. Aid F. (2013). Effects of dehydration and rehydration on the leaf lipids and lipid metabolism in *Parkinsonia aculeata* (Caesalpiniaceae). *Botany* 91 505–513. 10.1139/cjb-2013-0028

[B4] Blee E. (2002). Impact of phyto-oxylipins in plant defense. *Trends Plant Sci.* 7 315–321. 10.1016/s1360-1385(02)02290-2 12119169

[B5] Buerstmayr H. Krenn N. Stephan U. Grausgruber H. Zechner E. (2007). Agronomic performance and quality of oat (*Avena sativa* L.) genotypes of worldwide origin produced under Central European growing conditions. *Field Crops Res.* 101 343–351. 10.1016/j.fcr.2006.12.011

[B6] Chapman K. D. (1998). Phospholipase activity during plant growth and development and in response to environmental stress. *Trends Plant Sci.* 3 419–426. 10.1016/s1360-1385(98)01326-0

[B7] Conconi A. Miquel M. Browse J. A. Ryan C. A. (1996). Intracellular levels of free linolenic and linoleic acids increase in tomato leaves in response to wounding. *Plant Physiol.* 111 797–803. 10.1104/pp.111.3.797 12226331 PMC157897

[B8] de Ollas C. Hernando B. Arbona V. Gomez-Cadenas A. (2013). Jasmonic acid transient accumulation is needed for abscisic acid increase in citrus roots under drought stress conditions. *Physiol. Plant.* 147 296–306. 10.1111/j.1399-3054.2012.01659.x 22671923

[B9] Depaula F. M. Thi A. T. P. Zuilyfodil Y. Ferrariiliou R. Dasilva J. V. Mazliak P. (1993). Effects of water-stress on the biosynthesis and degradation of polyunsaturated lipid molecular-species in leaves of *Vigna unguiculata*. *Plant Physiol. Biochem.* 31 707–715.

[B10] Dong W. Lv H. Xia G. Wang M. (2012). Does diacylglycerol serve as a signaling molecule in plants? *Plant Signal. Behav.* 7 472–475. 10.4161/psb.19644 22499171 PMC3419036

[B11] Ehlers W. (1989). Transpiration efficiency of oat. *Agron. J.* 81 810–817. 10.2134/agronj1989.00021962008100050023x

[B12] Farooq M. Wahid A. Kobayashi N. Fujita D. Basra S. M. A. (2009). Plant drought stress: effects, mechanisms and management. *Agron. Sustain. Dev.* 29 185–212. 10.1051/agro:2008021

[B13] Franca M. G. C. Matos A. R. D’Arcy-Lameta A. Passaquet C. Lichtle C. Zuily-Fodil Y. (2008). Cloning and characterization of drought-stimulated phosphatidic acid phosphatase genes from *Vigna unguiculata*. *Plant Physiol. Biochem.* 46 1093–1100. 10.1016/j.plaphy.2008.07.004 18755595

[B14] Gasulla F. vom Dorp K. Dombrink I. Zaehringer U. Gisch N. Doermann P. (2013). The role of lipid metabolism in the acquisition of desiccation tolerance in *Craterostigma plantagineum*: a comparative approach. *Plant J.* 75 726–741. 10.1111/tpj.12241 23672245

[B15] Gigon A. Matos A. R. Laffray D. Zuily-Fodil Y. Pham-Thi A. T. (2004). Effect of drought stress on lipid metabolism in the leaves of *Arabidopsis thaliana* (ecotype Columbia). *Ann. Bot.* 94 345–351. 10.1093/aob/mch150 15277243 PMC4242175

[B16] Gong D.-S. Xiong Y.-C. Ma B.-L. Wang T.-M. Ge J.-P. Qin X.-L. (2010). Early activation of plasma membrane H+-ATPase and its relation to drought adaptation in two contrasting oat (*Avena sativa* L.) genotypes. *Environ. Exp. Bot.* 69 1–8. 10.1016/j.envexpbot.2010.02.011

[B17] Guy C. Kaplan F. Kopka J. Selbig J. Hincha D. K. (2008). Metabolomics of temperature stress. *Physiol. Plant.* 132 220–235. 10.1111/j.1399-3054.2007.00999.x 18251863

[B18] Hao Z. Liu X. Li X. Xie C. Li M. Zhang D. (2009). Identification of quantitative trait loci for drought tolerance at seedling stage by screening a large number of introgression lines in maize. *Plant Breed.* 128 337–341. 10.1111/j.1439-0523.2009.01642.x

[B19] Huws S. A. Kim E. J. Lee M. R. F. Scott M. B. Tweed J. K. S. Pinloche E. (2011). As yet uncultured bacteria phylogenetically classified as *Prevotella*, *Lachnospiraceae* incertae sedis and unclassified *Bacteroidales*, *Clostridiales* and *Ruminococcaceae* may play a predominant role in ruminal biohydrogenation. *Environ. Microbiol.* 13 1500–1512. 10.1111/j.1462-2920.2011.02452.x 21418494

[B20] Jeger M. J. Viljanen-Rollinson S. L. H. (2001). The use of the area under the disease-progress curve (AUDPC) to assess quantitative disease resistance in crop cultivars. *Theor. Appl. Genet.* 102 32–40. 10.1007/s001220051615

[B21] Kim E. H. Kim Y. S. Park S.-H. Koo Y. J. Do Choi Y. Chung Y.-Y. (2009). Methyl jasmonate reduces grain yield by mediating stress signals to alter spikelet development in rice. *Plant Physiol.* 149 1751–1760. 10.1104/pp.108.134684 19211695 PMC2663756

[B22] Kramer J. K. G. Zhou J. Q. (2001). Conjugated linoleic acid and octadecenoic acids: extraction and isolation of lipids. *Eur. J. Lipid Sci. Technol.* 103 594–600. 10.1002/1438-9312(200109)103:9<594::AID-EJLT5942>3.0.CO;2-R

[B23] Lee M. R. F. Tweed J. K. S. Moloney A. P. Scollan N. D. (2005). The effects of oil fish supplementation on rumen metabolism and the biohydrogenation of unsaturated fatty acids in beef steers given diets containing sunflower oil. *Anim. Sci.* 80 361–367. 10.1079/ASC41920361

[B24] Leshem Y. Y. Halevy A. H. Frenkel C. (1986). *Processes and Control of Plant Senescence.* Amsterdam: Elsevier Science Publishers B.V.

[B25] Løes A. K. Henriksen T. M. Eltun R. (2007). “N supply in stockless organic cereal production under northern temperate conditions. Undersown legumes or whole season green manure?,” in *Proceedings of the 3rd QLIF Congress: Improving Sustainability in Organic and Low Input Food Production Systems University*, Hohenheim, 230.

[B26] Marchin R. M. Turnbull T. L. Deheinzelin A. I. Adams M. A. (2017). Does triacylglycerol (TAG) serve a photoprotective function in plant leaves? An examination of leaf lipids under shading and drought. *Physiol. Plant.* 161 400–413. 10.1111/ppl.12601 28664534 PMC5877405

[B27] Mckersie B. D. Senaratna T. Walker M. A. Kendall E. J. Hethenngton P. R. (1988). “Deterioration of membranes during aging in plants: evidence for free radical mediation,” in *Senescence and Aging in Plants*, eds Noodén L. D. Leopold A. C. (San Diego, CA: Academic Press), 441–464.

[B28] Montilla-Bascón G. Sanchez-Martin J. Rispail N. Rubiales D. Mur L. Langdon T. (2013). Genetic diversity and population structure among oat cultivars and landraces. *Plant Mol. Biol. Report.* 31 1305–1314. 10.1007/s11105-013-0598-8 27524988

[B29] Mori I. C. Murata Y. Yang Y. Munemasa S. Wang Y.-F. Andreoli S. (2006). CDPKs CPK6 and CPK3 function in ABA regulation of guard cell S-type anion- and Ca2+-permeable channels and stomatal closure. *PLoS Biol.* 4:e327. 10.1371/journal.pbio.0040327 17032064 PMC1592316

[B30] Mossoba M. M. (2001). Analytical techniques for conjugated linoleic acid (CLA) analysis. *Eur. J. Lipid Sci. Technol.* 103:594 10.1002/1438-9312(200109)103:9<594::AID-EJLT5941>3.0.CO;2-U

[B31] Munnik T. Testerink C. (2009). Plant phospholipid signaling: “in a nutshell”. *J. Lipid Res.* 50 S260–S265. 10.1194/jlr.R800098-JLR200 19098305 PMC2674723

[B32] Nakashima K. Ito Y. Yamaguchi-Shinozaki K. (2009). Transcriptional regulatory networks in response to abiotic stresses in Arabidopsis and grasses. *Plant Physiol.* 149 88–95. 10.1104/pp.108.129791 19126699 PMC2613698

[B33] Nichols B. W. (1963). Separation of lipids of photosynthetic tissues - improvements in analysis by thin-layer chromatography. *Biochim. Biophys. Acta* 70 417–422. 10.1016/0926-6542(63)90060-x 14067615

[B34] Osakabe Y. Mizuno S. Tanaka H. Maruyama K. Osakabe K. Todaka D. (2010). Overproduction of the membrane-bound receptor-like protein kinase 1, RPK1, enhances abiotic stress tolerance in Arabidopsis. *J. Biol. Chem.* 285 9190–9201. 10.1074/jbc.M109.051938 20089852 PMC2838338

[B35] Osakabe Y. Osakabe K. Shinozaki K. Tran L.-S. P. (2014). Response of plants to water stress. *Front. Plant Sci.* 5:86. 10.3389/fpls.2014.00086 24659993 PMC3952189

[B36] Perlikowski D. Kierszniowska S. Sawikowska A. Krajewski P. Rapacz M. Eckhardt A. (2016). Remodeling of leaf cellular glycerolipid composition under drought and re-hydration conditions in grasses from the *Lolium festuca* complex. *Front. Plant Sci.* 7:1027. 10.3389/fpls.2016.01027 27486462 PMC4950141

[B37] Quartacci M. F. Glisic O. Stevanovic B. Navari-Izzo F. (2002). Plasma membrane lipids in the resurrection plant *Ramonda serbica* following dehydration and rehydration. *J. Exp. Bot.* 53 2159–2166. 10.1093/jxb/erf076 12379782

[B38] Ren C. Z. Ma B. L. Burrows V. Zhou J. Hu Y. G. Guo L. (2007). Evaluation of early mature naked oat varieties as a summer-seeded crop in dryland northern climate regions. *Field Crops Res.* 103 248–254. 10.1016/j.fcr.2007.07.001

[B39] Riemann M. Dhakarey R. Hazman M. Miro B. Kohli A. Nick P. (2015). Exploring jasmonates in the hormonal network of drought and salinity responses. *Front. Plant Sci.* 6:1077. 10.3389/fpls.2015.01077 26648959 PMC4665137

[B40] Sakaki T. Saito K. Kawaguchi A. Kondo N. Yamada M. (1990). Conversion of monogalactosyldiacylglycerols to triacylglycerols in ozone-fumigated spinach leaves. *Plant Physiol.* 94 766–772. 10.1104/pp.94.2.766 16667777 PMC1077297

[B41] Sakuma Y. Maruyama K. Qin F. Osakabe Y. Shinozaki K. Yamaguchi-Shinozaki K. (2006). Dual function of an *Arabidopsis* transcription factor DREB2A in water-stress-responsive and heat-stress-responsive gene expression. *Proc. Natl. Acad. Sci. U.S.A.* 103 18822–18827. 10.1073/pnas.0605639103 17030801 PMC1693746

[B42] Sánchez-Martín J. Heald J. Kingston-Smith A. Winters A. Rubiales D. Sanz M. (2015). A metabolomic study in oats (*Avena sativa*) highlights a drought tolerance mechanism based upon salicylate signalling pathways and the modulation of carbon, antioxidant and photo-oxidative metabolism. *Plant Cell Environ.* 38 1434–1452. 10.1111/pce.12501 25533379

[B43] Sánchez-Martín J. Mur L. A. J. Rubiales D. Prats E. (2012). Targeting sources of drought tolerance within an *Avena* spp. collection through multivariate approaches. *Planta* 236 1529–1545. 10.1007/s00425-012-1709-8 22824964

[B44] Sánchez-Martín J. Rispail N. Flores F. Emeran A. A. Sillero J. C. Rubiales D. (2017). Higher rust resistance and similar yield of oat landraces versus cultivars under high temperature and drought. *Agron. Sustain. Dev.* 37:3 10.1007/s13593-016-0407-5

[B45] Sánchez-Martín J. Rubiales D. Flores F. Emeran A. A. Shtaya M. J. Y. Sillero J. C. (2014). Adaptation of oat (*Avena sativa*) cultivars to autumn sowings in Mediterranean environments. *Field Crops Res.* 156 111–122. 10.1016/j.fcr.2013.10.018

[B46] Singh S. C. Sinha R. P. Hader D. P. (2002). Role of lipids and fatty acids in stress tolerance in cyanobacteria. *Acta Protozool.* 41 297–308.

[B47] Solovchenko A. E. (2012). Physiological role of neutral lipid accumulation in eukaryotic microalgae under stresses. *Russ. J. Plant Physiol.* 59 167–176. 10.1134/s1021443712020161

[B48] Stevens E. J. Armstrong K. W. Bezar H. J. Griffin W. B. Hampton J. G. (2004). “Fodder oats an overview,” in *Fodder Oats: A World Overview*, eds Suttie J. M. Reynolds S. G. (Rome: FAO), 1–9.

[B49] Stockinger E. J. Gilmour S. J. Thomashow M. F. (1997). Arabidopsis thaliana CBF1 encodes an AP2 domain-containing transcriptional activator that binds to the C-repeat/DRE, a cis-acting DNA regulatory element that stimulates transcription in response to low temperature and water deficit. *Proc. Natl. Acad. Sci. U.S.A.* 94 1035–1040. 10.1073/pnas.94.3.1035 9023378 PMC19635

[B50] Sukhija P. S. Palmquist D. L. (1988). Rapid method for determination of total fatty-acid content and composition of feedstuffs and feces. *J. Agric. Food Chem.* 36 1202–1206. 10.1021/jf00084a019

[B51] Thi A. T. P. Dasilva J. V. Mazliak P. (1990). The role of membrane-lipids in drought resistance of plants. *Bull. Soc. Bot. France Actual. Bot.* 137 99–114.

[B52] Tran L.-S. P. Urao T. Qin F. Maruyama K. Kakimoto T. Shinozaki K. (2007). Functional analysis of AHK1/ATHK1 and cytokinin receptor histidine kinases in response to abscisic acid, drought, and salt stress in *Arabidopsis*. *Proc. Natl. Acad. Sci. U.S.A.* 104 20623–20628. 10.1073/pnas.0706547105 18077346 PMC2154481

[B53] Umezawa T. Yoshida R. Maruyama K. Yamaguchi-Shinozaki K. Shinozaki K. (2004). SRK2C, a SNF1-related protein kinase 2, improves drought tolerance by controlling stress-responsive gene expression in *Arabidopsis thaliana*. *Proc. Natl. Acad. Sci. U.S.A.* 101 17306–17311. 10.1073/pnas.0407758101 15561775 PMC535404

[B54] Upchurch R. G. (2008). Fatty acid unsaturation, mobilization, and regulation in the response of plants to stress. *Biotechnol. Lett.* 30 967–977. 10.1007/s10529-008-9639-z 18227974

[B55] van Meer G. Voelker D. R. Feigenson G. W. (2008). Membrane lipids: where they are and how they behave. *Nat. Rev. Mol. Cell Biol.* 9 112–124. 10.1038/nrm2330 18216768 PMC2642958

[B56] Vrablik T. L. Watts J. L. (2012). Emerging roles for specific fatty acids in developmental processes. *Genes Dev.* 26 631–637. 10.1101/gad.190777.112 22474257 PMC3323873

[B57] Xiao B. Huang Y. Tang N. Xiong L. (2007). Over-expression of a LEA gene in rice improves drought resistance under the field conditions. *Theor. Appl. Genet.* 115 35–46. 10.1007/s00122-007-0538-9 17426956

