# Supplementary material for: Fatty Acid Profile Changes During Gradual Soil Water Depletion in Oats Suggests a Role for Jasmonates in Coping With Drought
Source: Front Plant Sci. 2018 Jul 31;9:1077. doi: 10.3389/fpls.2018.01077 (PMC6090161; doi:10.3389/fpls.2018.01077)
Supplement: Supplementary file 1 [file Table_1.PDF]

Supplemental material

Fatty acid profile changes during water stress in oats suggests a role for jasmonates in coping with drought

Javier Sánchez-Martín[1], Francisco J Canales-Castilla[1], *Diego Rubiales[1], Aurelio Gómez-Cadena[3], Vicent Arbona[3], Luis A. J. Mur[2], Elena Prats[1*].*

[1]CSIC, Institute of Sustainable Agriculture, Apdo. 4084, E-14080 Córdoba, Spain.
[2]Institute of Biological, Environmental and Rural Sciences, University of Aberystwyth, UK.
[3]Ecofisiologia i Biotecnologia Dpt. Ciències Agràries i del Medi Natural. Universitat Jaume I - Campus Riu Sec. E-12071 Castelló de la Plana. Spain

*For correspondence: Elena Prats
CSIC, Institute for Sustainable Agriculture, Apdo. 4084, E-14080 Córdoba, Spain.
Tel: +34 957499291
E-mail: elena.prats@ias.csic.es

Supplemental Table 1. Fatty acid profile indicating the fatty acids detected in oat in the different lipid fractions, i.e: polar (PF), mono and di-acylgliceride (MAG+DAG), triacylgliceride (TAG) and free fatty acid (FFA) fractions. t indicate that only traces not in all samples were detected.

Lipid fraction	PF	MAG+ DAG	TAG	FFA	Lipid fraction	PF	MAG+ DAG	TAG	FFA
C4					C18:1, t13,t14				
C6			t		C18:1, cis-9 (incl. t13 & 14)	x		t	t
C8					C18:1, t15 (&/or c10)	x	x	t	t
C10	x	x			C18:1, cis 11	x			
C11					C18:1, cis 12				
C12	x	x	x	x	C18:1, cis 13 (?)				
C13					C18:1, t16				
C14:0	x		x	x	C18:1, cis 14 (?)				
C14:1,trans9					C18:2 t,t isomer?				
C15 iso					C18:2, t9,t12				
C14:1,cis9					C18:1, cis 15(?)				
C15 ante					C19:0				
C15:0					C18:2 t9,c12?				
C16:0Ald					11t,15c,C18:2/isomers C18:2				
iso C16:0 (?)					C18:2, c9,c12	x	x	x	x
C15:1, cis10					9c,15c,C18:2				
C16 ante					C19:1				
Unid FA (?)		x			C18:3 n-6				
C16:0	x	x	x	x	C18:3 n-3	x	x	x	x
C16:1, trans 9					C20:0	x	x	x	x
C16:1 trans (3?)	x		t		9c,11t				
C17 iso	x	x	t		CLA isomers/C20:1 t11				
C16:1 cis 9					CLA (incl c11t13)				
C17 ante					10t,12c				
Phytanic iso 1					C20:1c9?				
Phytanic iso 2					C20:1, c11 (t,c/c,c)/C18:4				
Phytanic iso 3					C20:1 isomer/9c, 11c	x	x	t	t
C17:0					possibly 10c12c				
C16:2 ?					possibly 11c13c				
C18:0Ald					11t,13t /13t,15t?				
C17:1 a					t,t (6,8 to 10,12)				
C17:1 b					C21:0				
C17:1 c					C20:2				
C17:1,c10					C20:3, n-6				
C18 ante					C20:4, n-6				
C16:3,n-3					C22/C20:3,n-3	x	x	x	x
C18:0	x	x	x	x	C22:1 n-9				
C18:1, t4					C20:5,n-3				
C18:1, t5					C23				
C18:1, trans-6,7&/or 8					C22:2, n-6				
C18:1, trans-9					C24:0	x		t	x
C18:1, trans-10					C22:4,n-6				
C18:1, trans-11					C24:1	x			
C18:1, trans-12					C22:5,n-3				
C18:1, cis 6					C22:6,n-3				

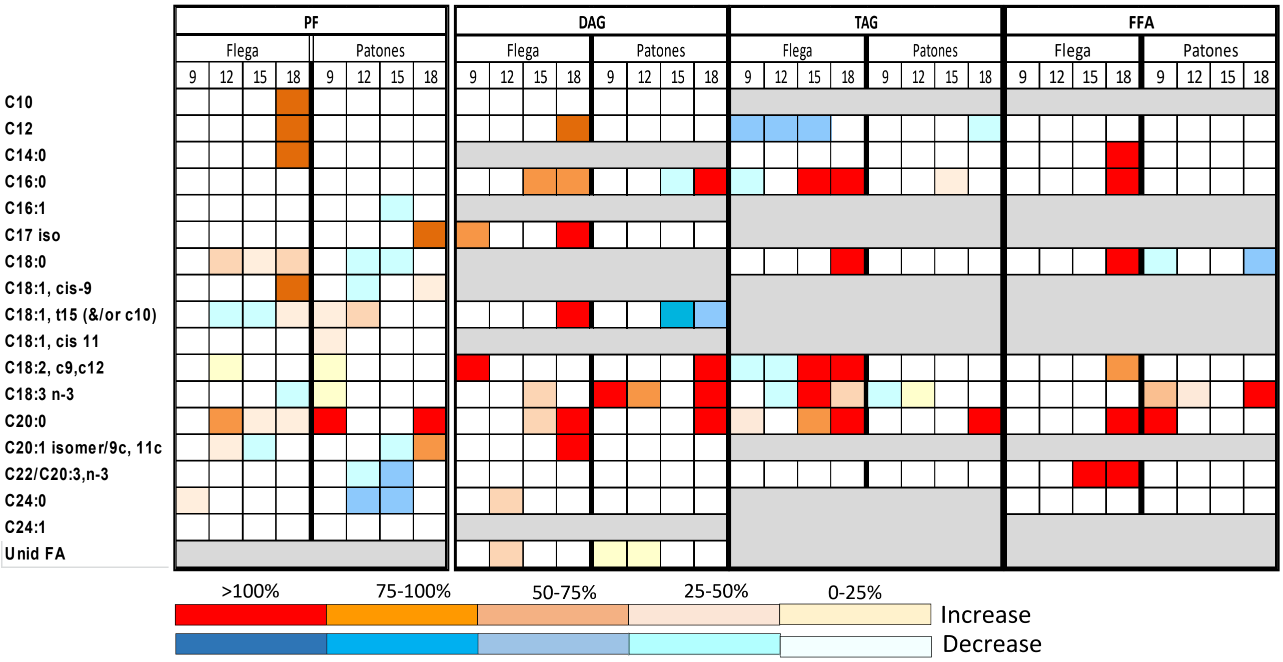

Supplemental Figure 1. Heat map of the fatty acid profile in the different lipid fractions. Color scale indicate increases or decreases of susceptible, Flega, and resistant, Patones, plants over a drought time course respect to their well watered controls. Grey sections indicate no detection of the corresponding fatty acid in that fraction
